# The association between intergenerational support and depressive symptoms in older adults: a chain mediation analysis of life satisfaction and well-being

**DOI:** 10.3389/fpubh.2025.1530639

**Published:** 2025-09-01

**Authors:** Wenjia Feng, Pengxin Geng, Yan Wang, Hongqing An, Qianqian Gao, Weiqin Cai, Qi Jing, Runguo Gao, Anning Ma

**Affiliations:** ^1^School of Public Health, Shandong Second Medical University, Weifang, China; ^2^School of Management, Shandong Second Medical University, Weifang, China; ^3^Institute of Public Health Crisis Management, Shandong Second Medical University, Weifang, China

**Keywords:** intergenerational support, depressive symptoms, life satisfaction, well-being, masking effect

## Abstract

**Background:**

With the acceleration of population aging, increasing attention has been directed toward mental health issues in later life. Among these, depressive symptoms represent one of the most prevalent psychological concerns and have been consistently associated with lower quality of life and impaired social functioning in older adults. As a primary source of social support for older adults, intergenerational support plays a crucial role in shaping their psychological well-being. This study aims to examine the potential mediating mechanisms linking intergenerational support to depressive symptoms among older adults, within the context of China's traditional culture of filial piety. The findings are intended to provide a theoretical basis for optimizing intergenerational support strategies and promoting mental health in later life.

**Methods:**

This study employed data from the 2020 wave of the China Family Panel Studies (CFPS), focusing on variables related to intergenerational support from children, life satisfaction, well-being, and depressive symptoms. Analysis methods included analysis of variance (ANOVA), Pearson correlation analysis, and bootstrap procedures to examine the chain mediation effects involving intergenerational support, life satisfaction, well-being, and depressive symptoms.

**Results:**

Emotional support (β = −0.431, *P* ≤ 0.001), life satisfaction (β_emotional support_ = −0.727, *P* ≤ 0.001; β_*economic support*_ = −0.757, *P* ≤ 0.001; β_*care support*_ = −0.756, *P* ≤ 0.001), and well-being (β_*emotional support*_ = −0.468, *P* ≤ 0.001; β_*economic support*_ = −0.518, *P* ≤ 0.001; β_*care support*_ = −0.504, *P* ≤ 0.001) were significantly associated with lower levels of depressive symptoms. Economic support (β =0.956, *P* ≤ 0.001) and care support (β =0.433, *P* ≤ 0.001) were positively associated with higher levels of depressive symptoms. Life satisfaction and well-being were found to exert a chain mediating effect in the association between intergenerational support and depressive symptoms [emotional support: total effect = −0.825, 95% CI (−0.990, −0.662); direct effect = −0.431, 95% CI (−0.5896, −0.2713); indirect effect = −0.0710, 95% CI (−0.0935, −0.0506); economic support: total effect = 0.7138, 95% CI (0.4609, 0.9667); direct effect = 0.9560, 95% CI (0.7185, 1.1936); indirect effect = −0.0373, 95% CI (−0.0664, −0.0106); care support: total effect = 0.2719, 95% CI (0.0061, 0.5377); direct effect = 0.4334, 95% CI (0.1836, 0.6832); indirect effect = −0.0289, 95% CI (−0.0587, −0.0016)].

**Conclusion:**

The findings reveal a chain mediation effect involving life satisfaction and well-being in the association between intergenerational support and depressive symptoms among older adults. Emotional support is positively associated with higher life satisfaction and greater well-being, which in turn are linked to lower levels of depressive symptoms. In contrast, life satisfaction and well-being appear to suppress the positive associations between economic support or care support and depressive symptoms. These results enhance our understanding of the psychosocial pathways through which intergenerational support is related to mental health in later life and provide empirical evidence to inform the design of targeted psychological interventions and social support policies.

## Introduction

Population aging has become a global trend (1). According to the Seventh National Population Census, individuals aged 60 and above account for 18.7% of China's total population, indicating that the country has entered a stage of advanced aging (2). With increasing life expectancy and a growing population of older adults, mental health concerns among older adults have received increasing attention. Studies have shown that approximately 38.6% of older Chinese adults exhibit depressive symptoms (3). These symptoms comprise a cluster of psychological problems—such as persistent sadness, loss of interest or pleasure, fatigue, self-deprecation, and suicidal ideation—that significantly affect individuals' emotions, cognition, and behavior, and interfere with daily functioning (4).

Chronic depression can seriously undermine individual's social support system, weakening connections with family, friends, and the broader community (5). As a core component of the social support network for older adults, intergenerational support is widely regarded as a critical determinant of their mental health [5]. Previous studies have demonstrated that intergenerational support can influence the mental health of older adults through multiple mechanisms (6–8). However, existing literature has predominantly focused on external social interaction pathways outside the family. For instance, Xu et al. (7) examined how intergenerational support affects mental health indirectly through older adults' attitudes toward the younger generation and their willingness to engage with them in community settings, highlighting the protective role of intergenerational communication among urban older populations. In contrast, the present study shifts the analytical lens inward to the psychological mechanisms within the family, emphasizing the internal pathways of intergenerational support under the context of traditional familial culture. Moreover, intergenerational support is inherently multidimensional, and different types of support may exert heterogeneous effects on mental health. For example, Morelli et al. (9) found that emotional support is generally associated with enhanced well-being and reduced risk of depression, whereas Wu (10) suggested that economic and instrumental support may increase psychological burden due to heightened dependency or shifts in family roles. Therefore, it is essential to further examine the mechanisms and pathways through which various types of intergenerational support affect depressive symptoms in later life.

Life satisfaction and well-being are dual indicators of mental health, reflecting cognitive evaluations and emotional experiences of life circumstances, respectively. Life satisfaction is typically shaped by structural factors such as economic security and living convenience (11, 12), whereas well-being is more closely related to intimate relationships and emotional connections (13, 14). Prior studies have shown that both life satisfaction and well-being are not only closely associated with depressive symptoms in older adults, but also serve as mediators in the relationship between intergenerational support and mental health outcomes (9, 15, 16). According to Diener's two-dimensional model of well-being, life satisfaction reflects an individual's overall cognitive judgment about life conditions, while well-being captures immediate emotional experiences (17). Stable and positive emotional support—such as care, attentive listening, and encouragement—can enhance older adults' sense of trust and security in interpersonal relationships, leading to a more favorable emotional appraisal of whether life is worthwhile, thereby increasing life satisfaction. In contrast, instrumental support—such as financial assistance or caregiving—may improve perceived living conditions in the short term, but its emotional effects depend heavily on the mode of support and the quality of interaction. Without accompanying emotional engagement, it may even evoke negative feelings of being “acted upon” or “dependent.” On this basis, emotional support is associated with greater life satisfaction, which may contribute to more positive emotional experiences. This interplay may form a reinforcing cycle that is linked to a lower risk of depressive symptoms.

Although existing theories offer possible pathway mechanisms, empirical research remains limited, particularly in systematically distinguishing the impact mechanisms of different types of intergenerational support. More importantly, the psychological impact of intergenerational support may vary significantly across cultural contexts. In Chinese society, where familial responsibility and intergenerational ethics are emphasized, support from children is not only a functional provision but also carries emotional and moral expectations. In the absence of emotional support, financial assistance may be perceived as insufficient or impersonal, potentially worsening the psychological condition of older adults (17).

Thus, this study aims to construct a chain mediation model incorporating life satisfaction and well-being as mediators to examine how different dimensions of intergenerational support (economic, emotional, and care) influence depressive symptoms among older adults. By clarifying the psychological transmission pathways of various support types, this research seeks to inform more targeted and culturally sensitive family-based interventions for late-life mental health. Based on these considerations, we propose the following hypotheses.

**Hypothesis 1:** intergenerational support influences depressive symptoms in older adults.
**Hypothesis 2:** life satisfaction mediates the relationship between intergenerational support and depressive symptoms.
**Hypothesis 3:** well-being mediates the relationship between intergenerational support and depressive symptoms.
**Hypothesis 4:** life satisfaction and well-being mediate the relationship between intergenerational support and depressive symptoms through a chain mediation model.

## Materials and methods

### Sample and data collection

**Figure 1:**
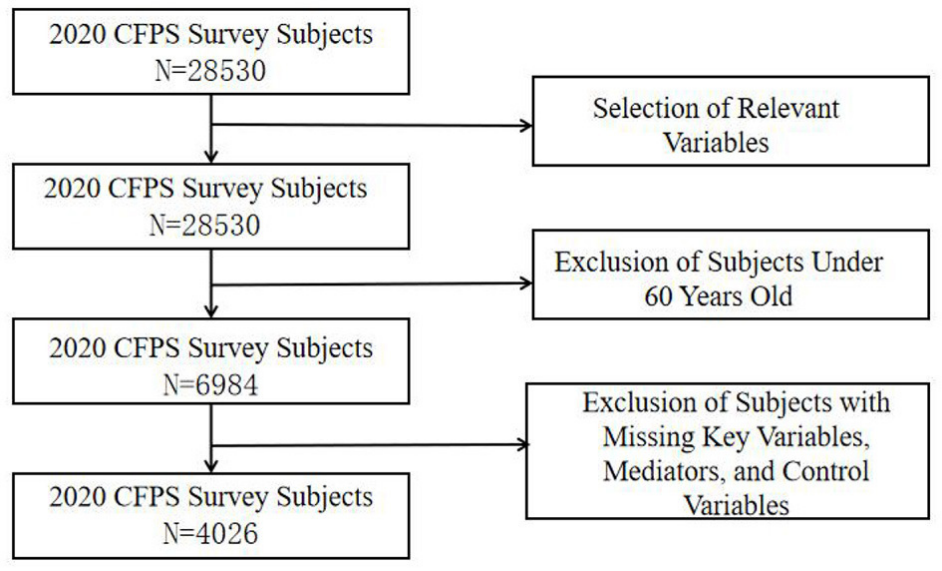
Variable screening process.

The data utilized in this study were obtained from the 2020 wave of the China Family Panel Studies (CFPS), a comprehensive social tracking survey conducted by the Institute of Social Science Survey at Peking University. CFPS systematically collects individual, household, and community data to reflect changes in China's society, economy, demographics, education, and health. Given that this study examines the impact of intergenerational relationships on depressive symptoms in older adults from a family perspective, the sample was restricted to individuals aged 60 and above who had marital experience and at least one child. After excluding cases with missing values for key variables, a final sample of 4,026 older adults with complete data was selected. The detailed selection process is illustrated in Figure 1.

### Measures

This study selects depressive symptoms as the dependent variable, intergenerational support from children as the independent variable, and life satisfaction and well-being as the mediating variables. Although the conceptual model is informed by the distinction between cognitive appraisal and emotional feedback, these constructs were not directly measured in the dataset. Instead, life satisfaction and well-being are used as proxy variables to represent these processes within the chain mediation model. The specific definitions of each variable are as follows:

**(1) Depressive symptoms**: The severity of depressive symptoms was assessed using the 8-item version of the Center for Epidemiologic Studies Depression Scale (CESD-8), which has demonstrated good reliability and validity among older adults in China (Cronbach's alpha = 0.8486) (18). Respondents were asked to rate the frequency of specific feelings and behaviors they experienced in the past week on a 4-point Likert scale ranging from 0 (“rarely or none of the time”) to 3 (“most or all of the time”). A sample item is “I felt depressed.” Positively worded items were reverse-coded. Total scores range from 0 to 24, with higher scores indicating more severe depressive symptoms.
**(2) Intergenerational support**: Building upon previous research (6–8, 10, 12), this study measures intergenerational support through caregiving (instrumental support), economic assistance (economic support), and emotional communication (emotional support). Instrumental support includes daily caregiving and household chores (9). Care support is assessed with the question: “In the past six months, have your children assisted you with household chores or daily care?” A response of 1 indicates care support, while 0 indicates no care support. Economic support is measured by asking, “In the past six months, have your children provided you with economic assistance?” with responses coded as 1 for economic support and 0 for no economic support. Emotional support is evaluated using the question: “How would you describe your relationship with your children over the past six months?” Responses range from “very distant” to “very close,” rated on a scale from 1 to 5.
**(3) Life satisfaction**: life satisfaction was assessed using the question from the CFPS database: “How satisfied are you with your life?” Responses were scored on a scale from 1 to 5, with higher scores indicating greater life satisfaction.
**(4) Well-being**: This variable was measured using the question from the CFPS database, “How happy are you?” Responses were rated on an integer scale from 0 to 10, with higher numbers indicating greater well-being.
**(5) Control variables**: Based on previous studies (6–8), this study controlled for the following variables: age (1 = “60–69 years,” 2 = “70–79 years,” 3 = “80–89 years,” 4 = “≥90 years”), gender (0 = “female,” 1 = “male”), pension status (0 = “no,” 1 = “yes”), self-rated health status (1 = “unhealthy,” 2 = “average,” 3 = “healthy,” 4 = “fairly healthy,” 5 = “very healthy”), and chronic illness status (In the past six months, have you been diagnosed with a chronic disease by a doctor? 1 = “yes,” 2 = “no”).

### Statistical analysis

This study employed SPSS 26.0 for data processing and analysis. Descriptive statistics for categorical variables were expressed as proportions, while continuous variables were reported as means ± standard deviations (M ± SD). One-way analysis of variance (ANOVA) was conducted to examine differences in depressive symptoms across various demographic characteristics. Pearson correlation analysis was used to assess relationships among variables, and chain mediation analysis was performed using Model 6 of the PROCESS 4.0 macro. After incorporating control variables, intergenerational support from children was set as the independent variable (*X*), depressive symptoms as the dependent variable (*Y*), and life satisfaction (M1) and well-being (M2) as mediating variables. The bootstrap resampling method (5,000 iterations) was employed to examine the mediation effects and to estimate the 95% confidence intervals. The significance of the direct, indirect, and total effects was determined by whether the confidence intervals excluded zero. The random seed was set to 12,345 before each analysis to ensure consistency and reproducibility. This approach follows the recommendation of Hayes (19), ensuring that identical bootstrapped samples are maintained across different models, thereby enhancing the stability of parameter estimates.

## Results

### Descriptive statistical analysis

This study included 4,026 older adults, with an average age of 68.09 ± 5.72 years. The average CES-D score was 4.99 ± 4.34. There were significant differences in CES-D scores among older adults of different genders, self-rated health statuses, chronic illness statuses, levels of emotional support, economic support, and care support (Table 1).

**Table 1:** Descriptive statistical analysis (*N* = 4026).

**Variable**	**Category**	***N* (%)**	***M* ±SD**	***F*-value**	***P*-value**	***η^2^***
Age	60~	2,586 (64.2)	5.07 ± 4.40	1.707	0.163	0.001
	70~	1,269 (31.5)	4.82 ± 4.16			
	80~	167 (4.1)	5.08 ± 4.72			
	≥90	4 (0.1)	1.75 ± 1.50			
Gender	Male	2,117 (52.6)	4.38 ± 4.09	89.170	<0.001	0.022
	Female	1,909 (47.4)	5.66 ± 4.50			
Pension	Yes	2,774 (68.9)	5.05 ± 4.35	1.830	0.176	<0.001
	No	1,252 (31.1)	4.85 ± 4.31			
Self-rated health	Unhealthy	970 (24.1)	7.52 ± 4.99	134.804	<0.001	0.118
	Average	688 (17.0)	4.87 ± 3.95			
	Fairly healthy	1520 (37.8)	4.27 ± 3.72			
	Healthy	423 (10.5)	3.41 ± 3.51			
	Very healthy	427 (10.6)	3.54 ± 3.68			
Chronic illness	Yes	1,180 (29.3)	6.06 ± 4.61	103.445	<0.001	0.025
	No	2,846 (70.7)	4.55 ± 4.14			
Emotional support	Very Distant	17 (0.4)	6.18 ± 5.87	36.465	<0.001	0.035
	Not Close	33 (0.8)	8.24 ± 6.33			
	Average	527 (13.1)	6.72 ± 4.87			
	Close	1,293 (32.1)	5.12 ± 4.33			
	Very Close	2,156 (53.6)	4.43 ± 4.01			
Economic support	Yes	2,148 (53.4)	5.41 ± 4.39	43.098	<0.001	0.011
	No	1,878 (46.6)	4.51 ± 4.23			
Care support	Yes	1,411 (35.0)	5.22 ± 4.25	6.452	0.011	0.002
	No	2,615 (65.0)	4.86 ± 4.38			

The 4,026 survey respondents were analyzed according to the scores of three entries: depressive symptoms, well-being, and life satisfaction. The mean score for depressive symptoms was (4.99 ± 4.34); the mean score for life satisfaction was (4.29 ± 0.85); and the mean score for Well-being was (7.79 ± 2.11) (Table 2).

**Table 2:** Scores for each item.

**Variable**	***N***	***M***	**SD**
Depressive symptoms	4,026	4.99	4.34
Life satisfaction	4,026	4.29	0.85
Well-being	4,026	7.79	2.11

### Pearson correlation analysis

Emotional support was positively correlated with life satisfaction and well-being scores (*P* < 0.01) and negatively correlated with depressive symptoms (*P* < 0.01); life satisfaction was positively correlated with well-being (*P* < 0.01) and negatively correlated with depressive symptoms (*P* < 0.01); and well-being was negatively correlated with depressive symptoms (*P* < 0.01) (Table 3).

**Table 3:** Correlation coefficient matrix for emotional support.

**Variable**	**Emotional support**	**Life satisfaction**	**Well-being**	**Depressive symptoms**
Emotional support	1.000			
Life satisfaction	0.166^**^	1.000		
Well-being	0.241^**^	0.441^**^	1.000	
Depressive symptoms	−0.177^**^	−0.291^**^	−0.346^**^	1.000

^**^Significant correlation at the 0.01 level (two-tailed).

Economic support is positively correlated with life satisfaction (*P* < 0.05) and positively correlated with both well-being and depressive symptoms (*P* < 0.01). Life satisfaction is positively correlated with well-being (*P* < 0.01) and negatively correlated with depressive symptoms (*P* < 0.01). Well-being is negatively correlated with depressive symptoms (*P* < 0.01) (Table 4).

**Table 4:** Correlation coefficient matrix for economic support.

**Variable**	**Economic support**	**Life satisfaction**	**Well-being**	**Depressive symptoms**
Economic support	1.000			
Life satisfaction	0.038^*^	1.000		
Well-being	0.082^**^	0.441^**^	1.000	
Depressive symptoms	0.104^**^	−0.291^**^	−0.346^**^	1.000

^*^Significant at the 0.05 level (two-tailed).

^**^Significant correlation at the 0.01 level (two-tailed).

Care support is positively correlated with both life satisfaction and depressive symptoms (*P* < 0.05) and positively correlated with well-being (*P* < 0.01). Life satisfaction is positively correlated with well-being (*P* < 0.01) and negatively correlated with depressive symptoms (*P* < 0.01). Well-being is negatively correlated with depressive symptoms (*P* < 0.01) (Table 5).

**Table 5:** Correlation coefficient matrix for care support.

**Variable**	**Care support**	**Life satisfaction**	**Well-being**	**Depressive symptoms**
Care support	1.000			
Life satisfaction	0.038^*^	1.000		
Well-being	0.058^**^	0.441^**^	1.000	
Depressive symptoms	0.040^*^	−0.291^**^	−0.346^**^	1.000

^*^Significant at the 0.05 level (two-tailed). ^**^Significant correlation at the 0.01 level (two-tailed).

### Analysis of chain mediation effects

This study conducted a multiple mediation analysis, with intergenerational support from children as the independent variable, depressive symptoms in older adults as the dependent variable, and life satisfaction and well-being as mediating variables while controlling for age, gender, pension status, self-rated health, and chronic disease prevalence. The analysis results indicated the following: In terms of emotional support, a positive correlation was found between emotional support and both life satisfaction (β = 0.1588, *P* < 0.001) and well-being (β = 0.4459, *P* < 0.001). In contrast, a negative correlation was observed with depressive symptoms (β = −0.4305, *P* < 0.001). Life satisfaction was positively associated with well-being (β = 0.9564, *P* < 0.001) and negatively associated with depressive symptoms (β = −0.7268, *P* < 0.001), while well-being was also negatively correlated with depressive symptoms (β = −0.4676, *P* < 0.001). Regarding economic support, significant positive correlations were identified between economic support and life satisfaction (β = 0.0714, *P* < 0.01), well-being (β = 0.291, *P* < 0.001), and depressive symptoms (β = 0.956, *P* < 0.001). Life satisfaction was positively correlated with well-being (β = 1.0086, *P* < 0.001) and negatively correlated with depressive symptoms (β = −0.7574, *P* < 0.001). In contrast, well-being also showed a negative association with depressive symptoms (β = −0.5182, *P* < 0.001). For care support, positive correlations were found between care support and life satisfaction (β = 0.0567, *P* < 0.05), well-being (β = 0.178, *P* < 0.01), and depressive symptoms (β = 0.4334, *P* < 0.001). Life satisfaction was positively correlated with well-being (β = 1.0127, *P* < 0.001) and negatively correlated with depressive symptoms (β = −0.7558, *P* < 0.001), while well-being also exhibited a negative correlation with depressive symptoms (β = −0.5036, *P* < 0.001).

According to the results of the multiple mediation model analysis, the direct effects of intergenerational support from children (emotional support, economic support, and care support) on depressive symptoms in older adults were statistically significant (95% CI: −0.5896 ~ −0.2713; 0.7185 ~ 1.1936; 0.1836 ~ 0.6832). The mediating effects of life satisfaction and well-being in the relationship between intergenerational support (emotional, economic, and care support) and depressive symptoms were also statistically significant (95% CI: −0.1565 ~ −0.0775, −0.2638 ~ −0.1577; 95% CI: −0.0966 ~ −0.0153, −0.2191 ~ −0.0877; 95% CI: −0.0875 ~ −0.0022, −0.1520 ~ −0.0257). Furthermore, the chain mediation effects of life satisfaction and well-being in the association between intergenerational support and depressive symptoms were statistically significant (emotional support: 95% CI: −0.0935 ~ −0.0506; economic support: 95% CI: −0.0664 ~ −0.0106; care support: 95% CI: −0.0587 ~ −0.0016) (Table 6).

**Table 6:** Multiple mediation model effect diagram.

**Variable**	**Emotional support**			**Economic support**			**Care support**		
	**Life satisfaction**	**Well-being**	**Depressive symptoms**	**Life satisfaction**	**Well-being**	**Depressive symptoms**	**Life satisfaction**	**Well-being**	**Depressive symptoms**
Emotional support	0.159^***^	0.446^***^	−0.431^***^						
	(−9.340)	(−11.561)	(−5.303)						
Economic support				0.0714^**^	0.2910^***^	0.956^***^			
				(−2.712)	(−4.9004)	(−7.891)			
Care support							0.057^*^	0.178^**^	0.433^***^
							(−2.056)	(−2.858)	(−3.401)
Life satisfaction		0.956^***^	−0.723^***^		1.0086^***^	−0.757^***^		1.013^***^	−0.756^***^
		(−27.010)	(−9.120)		(−28.3833)	(−9.562)		(−28.455)	(−9.482)
Well-being			−0.468^***^			−0.518^***^			−0.504^***^
			(−14.316)			(−16.146)			(−15.625)
Age	0.118^***^	0.231^***^	−0.063	0.112^***^	0.206^***^	−0.028	0.109^***^	0.198^***^	−0.050
	(−5.215)	(−4.513)	(−0.592)	(−4.878)	(−3.981)	(−0.265)	(−4.767)	(−3.821)	(−0.468)
Gender	0.012	−0.024	−1.066^***^	0.003	−0.042	−0.976^***^	0.002	−0.0500	−1.010^***^
	(−0.450)	(−0.403)	(−8.774)	(−0.124)	(−0.703)	(−8.059)	(−0.075)	(−0.839)	(−8.292)
Self-rated health	0.117^***^	0.2097^***^	−0.654^***^	0.133^***^	0.248^***^	−0.662^***^	0.132^***^	0.244^***^	−0.680^***^
	(−10.554)	(−8.294)	(−12.385)	(−11.987)	(−9.749)	(−12.637)	(−11.888)	(−9.556)	(−12.910)
Pension	0.071^*^	0.048	0.264^*^	0.072^*^	0.044	0.233	0.071^*^	0.044	0.240^*^
	(−2.517)	(−0.755)	(−2.022)	(−2.526)	(−0.682)	(−1.790)	(−2.507)	(−0.686)	(−1.836)
Chronic illness	0.023	0.096	0.782^***^	0.029	0.108	0.743^***^	0.028	0.107	0.747^***^
	(−0.768)	(−1.430)	(−5.606)	(−0.943)	(−1.577)	(−5.348)	(−0.919)	(−1.567)	(−5.340)
Constant	3.060^***^	1.430^**^	15.616^***^	3.686^***^	2.314^***^	13.707^***^	3.711^***^	2.416^***^	14.034^***^
	(−34.608)	(−3.543)	(−33.302)	(−67.203)	(−12.850)	(−36.672)	(−69.611)	(−13.522)	(−37.601)
*R* ^2^	0.062	0.238	0.225	0.044	0.217	0.232	0.043	0.214	0.222
*F*	44.593	179.123	146.066	30.696	159.233	151.536	30.152	156.358	143.413

(1) Standardized regression coefficients are shown in the table, and numbers in parentheses are *t*-statistics; (2) ^*^P < 0.05, ^***^P < 0.01, ^****^P ≤ 0.001.

**Figure 2:**
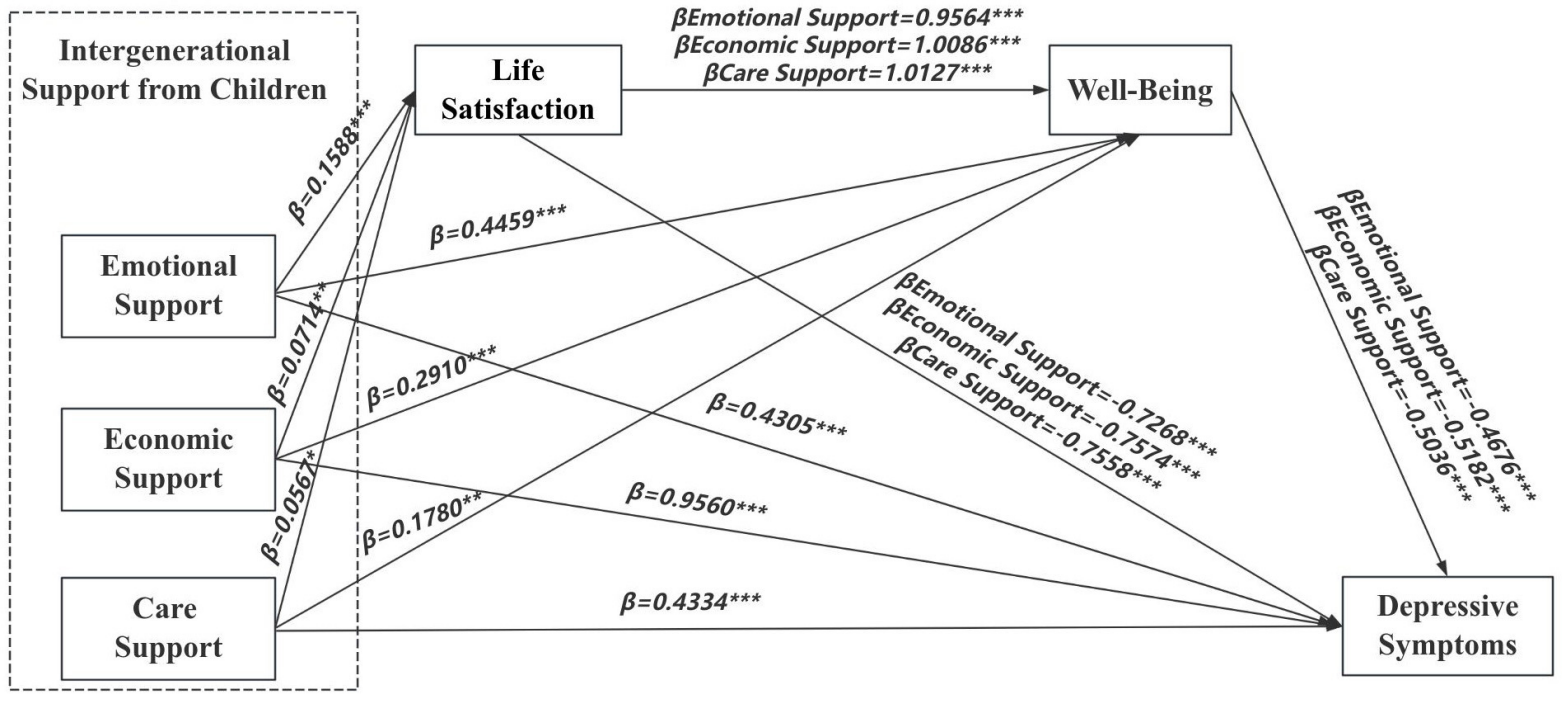
Mediation analysis model of intergenerational support from children and depressive symptoms in older adults. *P < 0.05, **P < 0.01, ***P ≤ 0.001.

The direct effect values of emotional, economic, and care support were −0.4305, 0.9560, and 0.4334, respectively. The total effect values were −0.8254, 0.7138, and 0.2719, respectively, while the total mediation effect values were −0.3949, −0.2422, and −0.1615. Therefore, all hypotheses were supported. Life satisfaction and well-being exhibited a masking effect in the relationship between economic/care support and depressive symptoms, where the direct and indirect effects had opposite signs, resulting in the total effect being masked. The results are presented in Table 7 and Figure 2.

**Table 7:** Significance test of mediation effects.

**Variable**	**Path**	**Boot SE**	**Effect**	**95% CI**
Emotional support	Direct effect	0.081	−0.431	−0.590 −0.271
	Total effect	0.083	−0.825	−0.989 −0.662
	Total mediation effect	0.037	−0.395	−0.468 −0.324
	Emotional support—Life satisfaction—Depressive symptoms	0.020	−0.115	−0.157 −0.078
	Emotional support—Well-being—Depressive symptoms	0.027	−0.209	−0.264 −0.158
	Emotional support—Life satisfaction—Well-being - Depressive symptoms	0.011	−0.071	−0.094 −0.051
Economic Support	Direct effect	0.121	0.956	0.719 1.194
	Total effect	0.129	0.714	0.461 0.967
	Total mediation effect	0.049	−0.242	−0.337 −0.147
	Economic support—Life satisfaction—Depressive symptoms	0.021	−0.054	−0.097 −0.015
	Economic Support - Well-being - Depressive Symptoms	0.034	−0.151	−0.219 −0.088
	Economic Support - Life Satisfaction - Well-being - Depressive symptoms	0.014	−0.037	−0.066 −0.011
Care support	Direct effect	0.127	0.433	0.184 0.683
	Total effect	0.136	0.272	0.006 0.538
	Total mediation effect	0.048	−0.162	−0.256 −0.067
	Care support—Life satisfaction—Depressive symptoms	0.022	−0.043	−0.088 −0.002
	Care support—Well-being—Depressive symptoms	0.032	−0.090	−0.152 −0.026
	Care support—Life satisfaction—Well-Being—Depressive symptoms	0.015	−0.029	−0.059 −0.002

## Discussion

This study utilized data from the 2020 China Family Panel Studies (CFPS) and conducted a cross-sectional study on older adults aged 60 and above. It analyzed the interrelationships and mechanisms of intergenerational support from children, life satisfaction, well-being, and depressive symptoms. The study found that life satisfaction and well-being played a chain mediation role in the relationship between intergenerational support from children and depressive symptoms among older adults.

### Intergenerational support influences depressive symptoms in older adults

Studies have shown that emotional support is negatively correlated with depressive symptoms in older adults, a finding consistent with numerous global studies indicating that closer relationships between children and their aging parents are associated with a lower risk of depression (20–23). According to the social convoy theory, family interactions provide a crucial support network for older adults (24). Increased emotional support enhances perceived social support, which in turn helps alleviate depressive symptoms (25).

In contrast, economic and care support are positively correlated with depressive symptoms, suggesting that such support may increase the risk of depression among older adults. This observation aligns with findings from studies conducted in Western countries (26–28). In China, political and economic reforms have transformed traditional family structures. While adult children have traditionally been obligated to obey and care for their parents, the younger generation has acquired higher-paying jobs and adopted more Western perspectives on eldercare, fostering more egalitarian parent-child relationships (29). Receiving economic support from children may lead aging parents to perceive a loss of economic independence, undermining their self-esteem and negatively impacting their mental health. Additionally, the erosion of traditional “filial piety” culture makes older adults increasingly aware of the burden placed on their children, fostering feelings of guilt and helplessness, thereby heightening the risk of depression.

Some scholars argue that care support can enhance interactions between older adults and family members, potentially reducing depressive symptoms (20, 21, 27, 30), which contradicts the findings of this study. Most respondents in this study were under the age of 70 (*n* = 3,855) or had no chronic illnesses (*n* = 2,846), making them more susceptible to “over-care” from their children. Relatively younger older adults with some degree of self-care ability may perceive excessive care support as intrusive, leading to psychological distress. This could undermine their perceived health status and self-efficacy or lead to intergenerational conflicts, potentially resulting in severe depression (31, 32).

Intergenerational support from children, particularly economic and care support, may contribute to dependence and family stress due to the redistribution of limited resources, ultimately negatively affecting the well-being of older adults. However, emotional support has been found to be more effective than economic and care support in enhancing the psychological well-being of aging parents, a finding consistent with Li's study (33). Therefore, it is crucial to encourage family members to prioritize the emotional needs and intimate relationships of older adults to mitigate their feelings of loneliness, isolation, and depression.

### Life satisfaction mediates the relationship between intergenerational support and depressive symptoms

This study reveals that life satisfaction mediates the relationship between intergenerational support from children and depressive symptoms among older adults. Life satisfaction is a fundamental determinant of psychological well-being, representing an individual's subjective assessment of overall life quality based on personal standards (34). Higher life satisfaction is associated with greater contentment with one's living conditions, a more positive mindset, better mental health, and lower levels of depression. Adequate intergenerational support significantly enhances life satisfaction among older adults (35, 36).

Emotional support facilitates the establishment of close family relationships, fulfilling mutual emotional needs among family members (37). Stronger emotional bonds enable individuals to feel valued, needed, and accepted within their families, enhancing their self-efficacy (34, 38). Furthermore, positive emotional relationships foster mutual understanding and trust, reducing intergenerational conflicts and improving family harmony, which contributes to higher life satisfaction among older adults (39, 40). As a result, emotional support alleviates negative emotions and lowers the risk of depression.

Economic support alleviates common economic burdens among older adults, such as medical, housing, and daily living expenses. This financial security fosters a sense of stability, enhancing their life satisfaction. Moreover, in traditional Chinese culture, the “reciprocity model” of family caregiving suggests that children's economic support reflects practical parental upbringing, reinforcing the older adults sense of accomplishment as parents, thus further boosting their life satisfaction (41).

Care support meets the daily needs of older adults, reducing their burdens and enhancing their overall life satisfaction. Additionally, caregiving provided by children embodies filial piety, aligning with traditional Chinese family caregiving norms, which bring a sense of comfort and validation to older adult parents (42). By receiving care support, older adults are better prepared for potential risks in later life, thereby improving their sense of life control and overall life satisfaction. Older adults with strong intergenerational support tend to experience higher life satisfaction and positive emotions, allowing them to assess their quality of life more favorably and cultivate a healthy social mindset (43).

### Well-being mediates the relationship between intergenerational support and depressive symptoms

The results indicate that well-being mediates the relationship between intergenerational support from children and depressive symptoms among older adults. Confucian filial piety is a fundamental aspect of traditional Chinese culture, and its influence remains significant in China. People widely acknowledge filial piety's familial obligations and responsibilities, which dictate that children should provide for, obey, and respect their aging parents (44). Under the influence of filial culture, older adults who receive intergenerational support from their children experience a sense of fulfillment, thereby enhancing their well-being (45).

First, emotional support from children helps older adults—who may feel marginalized due to changes in their social roles and advancing age—perceive their family as a strong support system, thereby improving their well-being (46). Second, economic support from children alleviates the economic burden on older adults, allowing them to invest in hobbies or purchase services from other institutions, thereby enhancing their well-being (47, 48). Finally, care support from children benefits older adults with mobility limitations or poor health by improving their daily well-being. As primary and essential caregivers, children significantly contribute to the well-being of older adults through their assistance (47, 49).

A greater sense of well-being fosters positive emotions among older adults, making them more likely to experience pleasure and satisfaction while reducing feelings of loneliness (50). Therefore, well-being plays a crucial role in improving mental health among older adults by reinforcing emotional, economic, and care support. It serves as a vital mediating variable between intergenerational support and depressive symptoms.

### Life satisfaction and well-being serve as a chain mediating effect between intergenerational support from children and depressive symptoms in older adults

Intergenerational support from children can enhance life satisfaction among older adults, thereby increasing their well-being and ultimately alleviating depressive symptoms. Life satisfaction represents an individual's overall evaluation of various aspects of life and is both a crucial component and a strong predictor of well-being (51). Older adults with high life satisfaction tend to experience greater well-being and fulfillment, and this positive emotional state helps them better cope with life's stresses and challenges (52). Well-being mitigates depressive symptoms by fostering positive emotions and a sense of social support while reducing loneliness and social isolation (53). Studies indicate that higher levels of well-being significantly improve mental health among older adults, decreasing anxiety and depressive symptoms (54). The analysis of economic and care support revealed a structural pattern in the chain mediation pathway, where the direct effects were positive while the indirect effects were negative—suggesting the presence of a “masking effect.” This indicates that although intergenerational support can indirectly alleviate depressive symptoms by enhancing life satisfaction and well-being, it may simultaneously impose certain psychological burdens. For example, while economic support provides material security, it may undermine older adults' sense of autonomy and independence, triggering feelings of being “acted upon” or indebted (29). Similarly, when care support exceeds actual needs, it may be perceived as “over-involvement,” diminishing the recipient's perceived sense of control (31). These emotional tensions may prevent older adults from fully benefiting psychologically from the material or caregiving support received, leading to opposing directions of effects within the mediation pathway.

### Implications

The following recommendations are proposed to enhance the mental well-being of older adults. First, emotional support should be strengthened, financial assistance optimized, and caregiving quality improved. Family members should be encouraged to engage in regular interactions through gatherings or activities to strengthen emotional bonds. Community-based family support groups should be established to facilitate experience sharing and provide guidance on better supporting older adults. Additionally, psychological counseling services should be provided to help family members better understand and meet the emotional needs of older adults (55).

When providing economic support, children should consult with older adults to ensure that the mode and amount of assistance are appropriate, thereby preventing financial strain and psychological distress. Older adults should be encouraged to engage in suitable economic activities or investments to enhance financial independence and improve life satisfaction (56). Furthermore, governments and society should strengthen the social security system for older adults by providing health insurance, pension plans, and other welfare programs to alleviate financial burdens (57).

When providing care support, the preferences and needs of older adults should be respected to avoid excessive intervention, thereby fostering autonomy and self-esteem. Specialized older adult care training should be provided to children to enhance their caregiving skills and improve the quality of care. Additionally, community-based caregiving services should be developed to assist older adults in need daily, thereby alleviating the caregiving burden on children (58).

Secondly, improving life satisfaction among older adults is essential. Participation in community activities, volunteer services, and interest groups should be encouraged to enhance social engagement and sense of belonging, thereby improving life satisfaction. A healthy lifestyle should be promoted by providing fitness, nutrition, and mental health guidance to help older adults maintain physical and psychological well-being. Traditional cultural activities should also be organized to promote filial piety and strengthen their sense of identity and fulfillment within the family and society (59).

Lastly, policy and societal support should be strengthened. Policies such as psychological counseling services and senior activity centers should be implemented to improve older adults' mental health and life satisfaction. A comprehensive social support network should provide multi-tiered and diverse assistance forms, enhancing older adults' life satisfaction and psychological well-being (60).

## Limitations

This study also has several limitations. First, this study is based on cross-sectional data from the 2020 CFPS, making it difficult to establish causal relationships among emotional, economic, and care support. Secondly, due to the limited scope of the CFPS, the extraction of information regarding the impact of intergenerational support on depressive symptoms among older adults was constrained. To ensure survey brevity and reduce respondent burden, this study employed single-item measures to assess the three dimensions of intergenerational support—economic, emotional, and care support. However, this simplification of complex, multidimensional constructs may have compromised the precision and accuracy of the measurements. Future research should incorporate multi-item scales (e.g., financial support covering amount, frequency, and satisfaction), behavioral records (e.g., actual expenditure data), or third-party reports (e.g., verification from family members) to comprehensively capture the multidimensional nature of intergenerational support and enhance the credibility of findings. Thirdly, in this study, chronic illness status was assessed as a binary variable (yes/no). While this approach allows for general control of health conditions, it does not account for the complexity of chronic diseases—such as the number of conditions, their severity, or their impact on daily functioning. Future research would benefit from a more comprehensive assessment of chronic illness, potentially incorporating multimorbidity indices or validated functional status scales, to enable a more nuanced understanding of how physical health interacts with intergenerational support and mental health outcomes. Finally, data on life satisfaction, well-being, and depressive symptoms were self-reported by respondents, which may have introduced recall and reporting biases. Future studies should employ longitudinal data spanning multiple years to understand better the causal relationships among emotional, financial, and care support and uncover intergenerational support's long-term effects on depressive symptoms in older adults. This would provide a more comprehensive and dynamic perspective for related research.

## Conclusion

The findings indicate that intergenerational support from children influences depressive symptoms among older adults. Furthermore, this study identifies that the intervention effect of intergenerational support on depressive symptoms in older adults is mediated through a sequential pathway involving life satisfaction and well-being. Specifically, emotional support enhances life satisfaction, increasing well-being and ultimately alleviating depressive symptoms. Financial and care support positively predict depressive symptoms among older adults. Policies should be implemented to enhance emotional support from children, as this is crucial for promoting active and healthy aging among older adults.

## Data Availability

The original contributions presented in the study are included in the article/supplementary material, further inquiries can be directed to the corresponding authors.
